# Healthcare professionals' perspectives on a blended care program in primary care; A qualitative study

**DOI:** 10.1016/j.invent.2021.100440

**Published:** 2021-08-04

**Authors:** S.A.J. Toonders, E.Y. Poolman, M.E. Nieboer, M.F. Pisters, C. Veenhof

**Affiliations:** a Department of Health Innovation and Technology, Fontys University of Applied Sciences, Eindhoven, the Netherlands; b Center for Physical Therapy Research and Innovation in Primary Care, Leidsche Rijn Julius Health Care Centers, Utrecht, the Netherlands; c Physical Therapy Research Group, Department of Rehabilitation, Physical Therapy Science and Sport, Brain Center Rudolf Magnus, University Medical Center Utrecht, Utrecht, the Netherlands; d Department of Human Movement Sciences, Faculty of Behavioural and Movement Sciences, Vrije Universiteit Amsterdam, Amsterdam, Movement Sciences, the Netherlands.; e Physical Therapy Sciences, program in Clinical Health Sciences, University Medical Center Utrecht, Utrecht University, the Netherlands; f Research Group Innovation of Human Movement Care, HU University of Applied Sciences, Utrecht, the Netherlands.

**Keywords:** GP, General Practitioner, MHN, Mental Health Nurse, MUPS, Medically Unexplained Physical Symptoms, PT, Physical Therapist, SUS, System Usability Scale, Usability, Medically unexplained physical symptoms, Blended therapy, E-coaching, Healthcare professionals

## Abstract

Increasingly, healthcare policies have changed focus from cure and care to behaviour and health. Prevention is becoming more important, which requires a change in the role of healthcare professionals. Healthcare professionals' role is changing from being a therapist to taking on the role of a coach. To prevent chronicity in Medically Unexplained Physical Symptoms (MUPS), an integrated blended care program was developed. To apply this new program in daily practice, it is important to gain insight into the usability. From the healthcare professionals' point of view the concept of usability consists of performance, satisfaction and acceptability. In this qualitative study participants were recruited after participating in the PARASOL program. Demographics were collected. Semi-structured interviews were conducted and analysed using thematic analysis. Ten healthcare professionals (six physical therapists and four mental health nurses) were interviewed. Four themes on usability were identified: (1) *Who fits in the program*, (2) *preparation*, (3) *experience with the program* and (4) *interprofessional collaboration*. This study gathered healthcare professionals' experiences with and attitudes towards integrating healthcare and offering blended care programs. An integrated blended care program offers the possibility to personalize treatment. Findings show attention should be given to the new responsibilities of healthcare professionals, and their role in integrated and blended care. This new approach of delivering healthcare can facilitate interprofessional collaboration. Achieving sustainable change in patients however still requires instruction and support for healthcare professionals implementing behavioural change techniques.

## Highlights

• Attention should be given to new responsibilities of healthcare professionals in their role in blended care
• The approach of offering integrated and blended care facilitates interprofessional collaboration
• Achieving sustainable change in patients requires support for professionals implementing behavioural change techniques

## 1 Introduction

Over 75% of the Dutch population visited the general practitioner (GP) in 2018 with an average of 4.5 visits per person per year (Meijer et al., 2019). About 30% of symptoms, e.g., pain, fatigue or dizziness (Trimbos-instituut, 2011) remain medically unexplained after patients visit their GP (Kroenke and Jackson, 1998; van Dessel et al., 2014). In most patients these symptoms disappear spontaneously after a few weeks. Nevertheless, for 2.5% of these patients, symptoms sustain and have a high impact on daily life (van Dessel et al., 2014; Verhaak et al., 2006) These so called Medically Unexplained Physical Symptoms (MUPS) are physical complaints that last for at least a few weeks, where no somatic condition is found that explains the complaints with adequate medical examination (Trimbos-instituut, 2011).

Providing appropriate treatment for people with MUPS at an early stage, with the use of neurosciences-based therapeutic education, cognitive behavioural therapy and exercise therapy which have been shown to be effective treatment modalities in patients with chronic MUPS, has multiple advantages (van Dessel et al., 2014). Literature shows effective outcomes on the reduction of unnecessary medical consumption, and increased job participation (Volker et al., 2015; Zeylemaker et al., 2015). MUPS can be divided into three consecutive stages, ranging from mild, to moderate to chronic stages. These stages are based on the frequency of consultations to the GP, duration of symptoms and experienced physical and/or psychological dysfunction (olde Hartman et al., 2009). Prevention in relation to MUPS seeks to identify individuals who show early signs of MUPS (van Westrienen et al., 2019).

In order to maintain healthcare accessibility and affordability, policy in the Netherlands has sought to change the way in which healthcare is organized. Moving from a focus on cure and care to behaviour and health (van Ewijk et al., 2013). This change requires a shift in healthcare delivery with more focus on prevention, from a traditional expert to a patient-centred approach (Hibbard, 2004). Therefore, the role of healthcare professionals also has to change, moving from focus on being a therapist to focus on being a coach (Wouters et al., 2018).

Recently, such an integrated blended care program to prevent chronicity in MUPS, the PARASOL program, has been developed in collaboration with healthcare professionals and patients (Van Westrienen et al., 2018). This specific program focuses on increasing insight into patients' perception of symptoms and modifiable prognostic risk factors for chronicity using therapeutic neuroscience education and encouraging self-management as well as an active lifestyle using a cognitive behavioural approach and graded activity (Van Westrienen et al., 2018). Blended care is the combination of online care and therapeutic guidance (Wentzel et al., 2016). The face to face sessions took place in the healthcare centre and lasted 30 min. Patients received 4 face-to-face sessions with the physical therapist (week 1, week 3, week 6 and week 12) where the focus was on the perception and acceptation of physical complaints. Patients received 3 face-to-face sessions with the mental health nurse (week 1, week 3, and week 6). In all 3 face-to-face sessions the mental health nurse was training coping strategies according to perpetuating factors and operant conditioning (Fordyce et al., 1973), with the focus on changing perception and acceptation (van Westrienen et al., 2018). Online care was provided using e-Coaching defined as ‘the of technology during coaching to motivate and stimulate (groups of) people to change attitudes, behaviours, and rituals’ (Lentferink et al., 2017; van Gemert-Pijnen et al., 2011). *E*-coaching provides information modules, personalized exercises and assignments to gradually increase the physical activity in a web based application and is not a standalone, but integrated in care. Online programs can not only be supportive of usual therapeutic guidance, but can also be a substantial element of the intervention as a whole (Erbe et al., 2017; Kloek et al., 2017). The combination of personal attention of a healthcare professional and the accessibility of an online tool is seen as highly promising, as it can stimulate patients to take an active role in their disease management (van der Vaart et al., 2014), as preparation can be done independently online and specific or substantive questions can be discussed at face-to-face meeting with professionals.

To implement a successful innovation, attention should be given to the unique position of end-users (van Gemert-Pijnen et al., 2011). The involvement of end-users provides direction for the development of integrated blended care programs. Co-creation, the engagement of users throughout the development process, is an important strategy in order to meet the values and needs (Craig et al., 2010). The objective of this study is to gain insight into the concept of usability, consisting of performance, satisfaction and acceptability from the healthcare professionals' perspective. Usability refers to ‘the quality of a system with respect to ease of learning, ease of use, user satisfaction and needs to be tested subjectively, from end-users perspective’ (De Bleser et al., 2011).

## 2 Methods

A qualitative design was chosen. Data were collected through semi-structured interviews with healthcare professionals recruited after participating in the clinical trial PARASOL (Van Westrienen et al., 2018).

### 2.1 PARASOL program

**Fig. 1:**
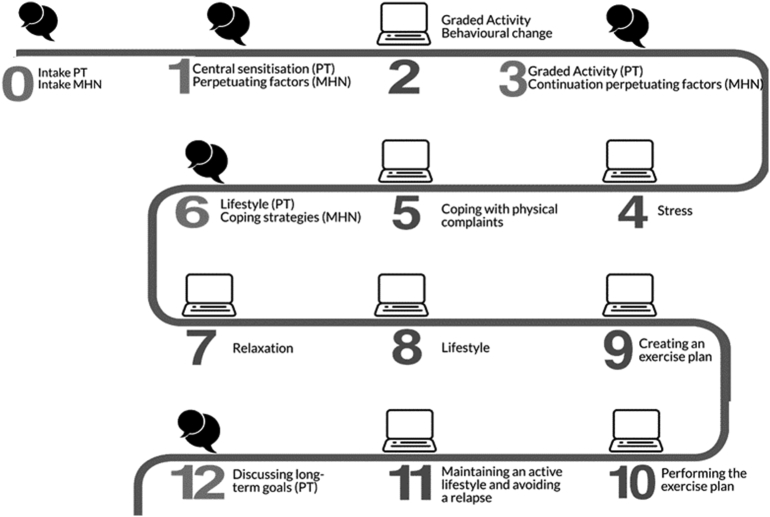
Overview of the PARASOL program. The text cloud indicates the face-to-face contact with a physical therapist (PT) and/or mental health nurse (MHN), the computer image indicates e-Coaching. The numbers represent the number of the week the related sessions were aimed.

The PARASOL program is a protocolled 12-week integrated blended care program. The program consists of five face-to-face consultations with a physical therapist and four sessions with a mental health nurse in primary care, supplemented with e-Coaching (Fig. 1) (Van Westrienen et al., 2018). Physical therapists and mental health nurses received instructions about the program during a two-day training session. These instructions included presentations on the study population, central sensitization, therapeutic neuroscience education, graded activity, and perpetuating factors (Van Westrienen et al., 2018). Furthermore, professionals were instructed on how to integrate *E*-coaching. All healthcare professionals received a protocol. Three months after the two-day training the PARASOL program started.

The e-Coaching modules consisted of information modules and videos on self-management and educative themes, videos and instructions on prescribed home exercises and assignments to gradually increase physical activity. Content was directed at patients' perception of symptoms, and modifiable prognostic risk factors for chronicity using therapeutic neuroscience education and encouraging self-management as well as an active lifestyle using a cognitive behavioural approach and graded activity. The e-Coaching modules complemented face-to-face treatments in order to introduce general themes, while during the contact with healthcare professionals, treatment could be personalized. Furthermore, during face-to-face treatment patients could pose questions to healthcare professionals. The basic functionality of e-Coaching used is based on the blended exercise intervention for patients with hip or knee osteoarthritis, called e-Exercise (Kloek et al., 2018).

### 2.2 Sample

Convenience sampling was used whereby the inclusion criterion was that healthcare professionals were eligible if they were involved in the PARASOL trial (seven physical therapists and six mental health nurses). All were approached to participate by the researcher (ST). We expected saturation at a sample size of eight to ten participants, based on similar published literature (Patel et al., 2020). Instructions were given by phone, information was sent by email and an appointment was made. Subsequently, informed consent was obtained.

### 2.3 Data collection

At the start of the semi-structured interview, demographic data such as age, gender, profession, work experience, number of patients treated in the PARASOL program and the System Usability Scale (SUS) score were collected. Semi-structured interviews were conducted by ST. A second researcher was present for non-verbal observation and verified if all questions were asked. The interview guide was based on the theoretic construct of De Bleser et al., 2011, offering direction to the interviews (De Bleser et al., 2011). This construct test existing electronic monitoring devices and divides criteria in objective and subjective dimensions. This study focused on the subjective dimension, containing user performance, satisfaction and acceptability (De Bleser et al., 2011). The interview guide was supplemented by determinants of healthcare innovation selected and developed by TNO (Netherlands Organisation for Applied Scientific Research) (Fleuren et al., 2014). After the first interviews were conducted, the interviewer added questions based on topics that emerged from previous interviews (for example ‘*How is your interest in technology in general?*’ and ‘*For which patients would this program be suitable?*’). The SUS consists of ten questions about the usability of a system (Brooke, 1996). The questions were answered on a numeric rating scale with a score range of one to five. A score of one stands for ‘strongly disagree’ and a score of five stands for ‘strongly agree’. The validated classification of the SUS of <70, a score between 70 and 80 or a score >80 respectively represent low, medium and high user usability. The SUS has a high reliability (α = 0.911) (Bangor et al., 2008). SUS scores were collected before the interview started and give information on the extent to which usability varies.

### 2.4 Data analysis

Interviews were recorded and transcribed verbatim and the audio interviews were checked by two researchers (ST & EP). Within one week after completing the interview, a brief summary was sent to all participants to ensure all information was interpreted correctly from the transcript. Thematic analysis was conducted (Braun and Clarke, 2006), whereby inductive codes were assigned to quotations that were related to the research question. Data were analysed manually and independently by two researchers (ST & EP). During the initial process of coding, transcripts were analysed line by line allowing the data to be fractured. These codes were highlighted and labelled within the text. During the axial coding process, fragments were put together. These fragments were categorized according to their similarities, after which main themes emerged, which were described and discussed by the researchers (ST, EP & MN). Finally, within the themes factors were labelled whether they were a facilitator or a barrier.

### 2.5 Validity

Validity was increased by creating a non-judgmental atmosphere in an independent position during the interviews and emphasizing the need to learn from the healthcare professionals. Fully transcribing the interviews decreases the chance of information bias, hence increasing accuracy and precision of the data collection that follows the interviews. The involvement of more than one researchers in collecting and analysing data increased the validity. Furthermore, the interpretation of the given answers was checked by the member check.

### 2.6 Ethical considerations

The study was approved by the Medical Ethical Committee Utrecht, by number 17/391. The dataset, including the interview guide, used and analysed during the current study are available from the corresponding author on reasonable request.

## 3 Results

Among the healthcare professionals contacted (seven physical therapists and six mental health nurses), two refused participation because they were not interested. One healthcare professional did not respond. In total ten healthcare professionals (six physical therapists and four mental health nurses) were interviewed. Of the ten participants, eight were female. Participants ranged in age from 25 to 62 years with a mean age of 35 years. Work experience ranged between 1.5 and 34 years. The number of patients treated with the PARASOL program ranged from 6 to 17. SUS scores ranged from 30 to 82.5, which implies eight healthcare professionals scored a low usability score, one a medium score and one healthcare professional scored a high usability score (Bangor et al., 2008). The interviews lasted between 30 and 45 min.

Besides demographics, questions about previous experiences with blended care (interest in blended care, expectations of integrated blended care programs) were asked. Most healthcare professionals volunteered to participate in this study out of personal interest in the subject matter. Although not everyone had previous experience with blended care, expectations of the blended program were cited as something new that fitted them well, and seen as the future of primary care. Although the healthcare professionals were optimistic about blended care, some were also afraid that the online program would take over their jobs. Through questions about usability from healthcare professionals' point of view, facilitators and barriers to implement an integrated blended care program were found. These are summarized in Table 1.

**Table 1:** Summary of facilitators and barriers linked to the (sub) themes.

Core theme	Facilitator	Barrier
Who fits in the program?	Intrinsic motivation of patients	Chronicity of complaints
Preparation	The use of a protocol	Duration between training and doing
	The number of patients treated	
Experience with the program	Achieving in depth treatment within the duration of a face-to-face session	Absence of evaluation time at the end of a program
		Difficulties setting (long-term) goals
		Difficulties in delivering care remotely, like e-Coaching
	Quality and structure of content	Technical issues
	Providing information interactively (text and video)	
Interprofessional collaboration	Holistic approach	Lack of feedback or confirmation from other disciplines
	Support of colleagues	

As seen in Table 1, the analysis resulted in four core themes. The themes are presented according to the sequence of the integrated blended care program.

### 3.1 Who fits in the program?

In the interviews, multiple situations were reported that hindered or favoured participation of patients in this integrated blended care program. The fact that the patient population strongly varied was repeatedly mentioned. Interviewees felt this integrated blended care program was not suitable for all included patients, specifically patients suffering from MUPS for a long time. Those interviewed felt that the intrinsic motivation of patients plays an important role in successfully completing the program. When describing their motivation, interviewees divided patients into roughly two groups; those motivated and those not motivated. Expectations of the study diverged between both groups. Motivated participants were well prepared and knew that the program had a low intensity and included guidance from a distance. Interviewees felt the outcomes for those less motivated were less positive, as they did not have a goal they could or wanted to work towards. ‘*If patients are too unprepared…I won't say they're less motivated, but they see themselves less as a problem owner* (p2)’. Although these patients were perhaps less motivated, healthcare professionals did not see it as a significant problem, as they expected less motivated patients to drop out at the start of the program. ‘*The people that aren't motivated, they'll drop out, they filter themselves out of the program* (p3)’.

### 3.2 Preparation

Before the start of the program, healthcare professionals had to attend a two day training in order to treat patients following the protocol of PARASOL independently. Some professionals learned a lot during those days, while others felt they knew sufficient about the subject matter at hand. A downside which was expressed was the long period between the introductory training and the start of the first treatment in the program. During the training days, an instruction protocol was handed out. Healthcare professionals used this protocol in different ways, as some mentioned they followed the protocol strictly. Others stuck less to the protocol. ‘*I never try to just plainly follow it, because then you lose contact with what is happening on the other side* (p3)’. Furthermore, the number of patients treated was mentioned as an important factor to make the program their own. Interviewees expressed that as they treated more patients, they better mastered the program. ‘*You can only make it your own if you see a lot of patients in a row* (p6)’.

### 3.3 Experience with the program

The interviewed healthcare professionals were generally positive about the integrated blended care program. A positive point highlighted was healthcare professionals noticed that patients became more aware of their responsibility for their own health. Patients became more self-managing of their problems. ‘*They really become problem owner!* (p2)’. As patients started the program at home, they found that patients were better prepared. This made the healthcare professionals able to get to the core of the treatment faster. ‘*Part of what is told, is already told online. The patient can see and read it himself. That saves time during treatment.* (p3)’ ‘*I notice patients learn a lot when they read material at home or watched a video* (p9)’. It was a unique experience, which relieved the workload and should therefore be implemented in usual care: ‘*… if people return, they changed something and are enthusiastic and proud about that. That they reached goals they didn't expect to* (p5)’. Overall, there was satisfaction with the session time of 25–30 min. Only during intake this was experienced as too short. Interviewees suggested doubling the time during intake to gain a wider picture of the patient. Concerning the treatment frequency of the program, the main point put forward was the need for more evaluation moments. Interviewees wanted to know what the program had meant for their patients. ‘*I just give a bunch of information to the patient, but have no clue whether it sticks with them* (p2)’. In some cases, patients did not have any questions for the healthcare professionals. This made it hard for them to know if there was sufficient commitment. ‘*It's a bit indecipherable* (p4)’. Healthcare professionals then struggled to formulate long-term goals with their patients. In terms of content, the information modules were perceived as well written and structured. Patients were given information in different ways (reading online, watching instruction videos) causing the information to stick better, as well as stimulating self-management among patients, which reduced healthcare professionals' workload. ‘*Texts were written in such a way* (…) *that people recognize themselves in it, they don't put off people* (p7)’. Besides, ‘*It is important that people get to process information in different ways, as our brain doesn't work like:* ‘*hi, let's change something*’*. So that's really necessary* (p2).’ There were also a number of criticisms regarding the accessibility of the e-Coaching modules. It was mentioned that there were many technical complications, such as difficulties with logging in and useless buttons. The professionals expressed doubts as to whether the e-Coaching application can offer functionality. ‘… *I wouldn't accept it if I couldn't log in* (…) *then I would really ask my money back* (p2)’. Healthcare professionals sought to deal with the technical defects as good as possible. Some printed exercises and others emailed them to the patients, enabling patients to still follow the program.

### 3.4 Interprofessional collaboration

One of the main added values mentioned was interprofessional collaboration between physical therapists and the mental health nurses induced by the integrated blended care program. Before the start of this program, it seemed as though healthcare professionals did not actively seek collaboration. ‘*I got to know the mental health nurse through this project* (p8)’. Through working together in this program, professionals better found each other. Contacts between professionals were easy to establish. Nearly all participants found the collaboration pleasant, helpful and experienced it as adding value because of the holistic approach. ‘*She sees things that I do not. I see things that she does not* (p1)’. ‘*That you seek cooperation, but stay within your own field* (p6)’. ‘*The added value is in the coordination* (p4).’ After the treatment was finished, most professionals continued to collaborate. They mentioned consulting each other more often. During the program there was little support or contact with the general practitioner (GP). This was not mentioned as being problematic, yet some interviewees indicated some feedback or confirmation by the GP would have been nice for reassurance. Support of colleagues was experienced as motivating and stimulating.

## 4 Discussion

This qualitative study was conducted to investigate the usability of an integrated blended care program from healthcare professionals' perspective. Semi-structured interviews were held, out of which four core themes emerged with accompanying facilitators and barriers.

The main facilitator in this integrated blended care program was the depth in treatments and the possibility to personalize the program. Patients received information in multiple ways and in different stages of the program. Prior to the face-to-face treatment, patients received information in text and saw instruction videos at home. Later, in face-to-face appointments with healthcare professionals, they could ask their questions or share their doubts. Patients were better prepared about what was going to happen next. Furthermore, repeating information made patients better prepared which saved time and allowed healthcare professionals to move on to the core of treatment faster. Repetition is a known behavioural change technique, as with repetition individuals better develop skills to actively self-regulate their behaviour (Kwasnicka et al., 2016). Another facilitator was the presence of two different types of healthcare professionals which led to a more holistic treatment. The two professions worked from their own vision, making the treatment as thorough as possible. After the program was finished, professionals had better gotten to know each other, and were actively seeking collaboration. It is remarkable that the collaboration picked up so fast, as literature shows interprofessional collaboration between healthcare professionals is complex (D'Amour et al., 2005). Professionals have their own educational background and are socialized to adopt a discipline-based vision of their patients and the services they offer. Collaboration requires making changes to this paradigm (D'Amour et al., 2005), which apparently succeeded in this blended treatment.

Participating healthcare professionals did not have the feeling all patients were suited to participate in the program and could be seen as an important barrier. This could be due to the condition of MUPS, which is hard to define and does not have clear criteria (Landelijke Stuurgroep Multidisciplinaire Richtlijnontwikkeling in de GGZ, 2010). Healthcare professionals could quickly tell if a patient was motivated or not, which they seemed to find a predictor of succeeding with the program or not. It seems required to first invite patients to share their motivations, personal needs and preferences before starting an integrated blended care program (Wentzel et al., 2016). Patients were selected through a proactive approach. An electronic screening method using data from the electronic medical record of the patients' GP was used (Van Westrienen et al., 2019). All eligible patients who were at risk for chronicity of complaints were proactively approached by their GP via an invitation letter explaining the study. By approaching patients proactively, the chance of finding patients who may be less motivated and less clear about what they want to achieve within the intervention may increase. One should therefore take motivation and personal help-request into account in future programs.

The most frequently reported barrier in the application of the integrated blended care was dealing with the autonomy regarding decisions about when and how to stick to the treatment protocol. For instance, professionals felt more time was needed during the intake, felt the need for an evaluation or booster session, and experienced the need for treating more patients following the protocol. Additionally, healthcare professionals struggled with the fact that their role changed into being more of a coach. They had difficulties seeking to formulate long terms goals with their patients. The feeling was patients did not have a specific help-request. This could be due to fact patients were better prepared. Furthermore, this preventative approach was new, which was hard to get used to. More insights are necessary into how to coach professionals on behavioural change techniques and how to organize healthcare around it (Hibbard, 2004; Talboom-Kamp et al., 2018; Wouters et al., 2018).

Other perceived barriers included the lack of accessibility of the e-Coaching modules, which was also reflected in by the reported SUS scores. Eighty percent of interviewees gave SUS scores below 70, which implies a low user satisfaction (Brooke, 1996). Technical problems were experienced as hindering factor, and the need for user-friendly technical solutions has been repeatedly expressed in the literature (Alkhaldi et al., 2014; Andersson et al., 2016; Kivi et al., 2015). The successful implementation of the integrated blended care will certainly require a more sophisticated technical setup, that is free of typical starting problems. Based on the results of the current study, a new application was developed which shows promising technical support.

### 4.1 Limitations and strengths

The main limitation of this study is that not all healthcare professionals who participated in the PARASOL program were included. It is possible that the professionals who were less satisfied did not participate. This gives a possible influence on the results. Another limitation is that the PARASOL program is the first program conducted at patients who suffer with moderate MUPS. A major advantage is that we can now gain insight into the first insights, but it remains difficult to make a comparison with existing literature, which focuses on chronic MUPS. Besides the fact that this study offers new insights into the end-user experience, the strengths of these studies are focused on the presence of the iterative analysis process and the triangulation in data collection and analysis. In this study, we applied two frameworks (De Bleser and TNO) to gain a broad perspective on both user experience and innovations in healthcare (De Bleser et al., 2011)(Fleuren et al., 2014). Although these frameworks guided on the researcher in data collection, the risk of bias was limited by triangulating in data collection and analysis.

## 5 Conclusion

An integrated blended care program offers the possibility to personalize treatment. This study gathered healthcare professionals' experiences with and attitudes towards integrating healthcare and offering blended care programs. Findings show attention should be given to the new responsibilities of healthcare professionals, and their role in integrated and blended care. This new approach of delivering healthcare can facilitate interprofessional collaboration. Achieving sustainable change in patients however still requires instruction and support for healthcare professionals implementing behavioural change techniques.

## Declarations

### Ethics approval and consent to participate

The study was approved by the Medical Ethical Committee Utrecht, by number 17/391. Informed consent was obtained from all individual participants included in the study.

### Consent for publication

Not applicable.

### Availability of data and materials

The datasets used and analysed during the current study are available from the corresponding author on reasonable request.

### Declaration of competing interest

The authors declare that they have no competing interests.

### Funding

This work was supported by SIA-RAAK-public [Grant No. 2015-02-24P].

### CRediT authorship contribution statement

ST, MP en CV initiated this study and contributed to the concept and design of this study. ST organized and participated as interviewer and EP participated as observer. ST en EP analysed and interpreted the data. MN conducted the role of qualitative expert. All authors revised the manuscript and approved the final version for submission.

### Acknowledgements

Not applicable.

## References

[bb0005] Alkhaldi B., Sahama T., Huxley C., Gajanayake R. (2014). Barriers to implementing eHealth: a multi-dimensional perspective. Stud. Health Technol. Inform..

[bb0010] Andersson G., Topooco N., Havik O., Nordgreen T. (2016). Internet-supported versus face-to-face cognitive behavior therapy for depression. Expert. Rev. Neurother..

[bb0015] Bangor A., Kortum P.T., Miller J.T. (2008). An empirical evaluation of the system usability scale. Int. J. Hum. Comput. Interact..

[bb0020] Braun V., Clarke V. (2006). Qualitative research in psychology using thematic analysis in psychology using thematic analysis in psychology. Qual. Res. Psychol..

[bb0025] Brooke J. (1996). SUS - a quick and dirty usability scale. Usability Evaluation in Industry.

[bb0030] Craig P., Dieppe P., Macintyre S., Michie S., Nazareth I., Petticrew M. (2010). Developing and evaluating complex interventions: the new Medical Research Council guidance. Evidence-Based Public Health: Effectiveness and Efficiency.

[bb0035] D’Amour D., Ferrada-Videla M., San Martin Rodriguez L., Beaulieu M.D. (2005). The conceptual basis for interprofessional collaboration: Core concepts and theoretical frameworks. J. Interprof. Care.

[bb0040] De Bleser L., De Geest S., Vincke B., Ruppar T., Vanhaecke J., Dobbels F. (2011). How to test electronic adherence monitoring devices for Ise in daily life: a conceptual framework. Comput. Inform. Nurs..

[bb0045] Erbe D., Eichert H.-C., Riper H., Ebert D.D. (2017). Blending face-to-face and internet-based interventions for the treatment of mental disorders in adults: systematic review. J. Med. Internet Res..

[bb0050] Fleuren M.A.H., Paulussen T.G.W.M., van Dommelen P., van Buuren S. (2014). Measurement instrument for determinants of innovations (MIDI). Int. J. Qual. Health Care.

[bb0055] Fordyce W., Fowler R., Lehmann J., Delateur B., Sand P., Trieschmann R. (1973). Operant conditioning in the treatment of chronic pain. Arch. Phys. Med. Rehabil..

[bb0060] Hibbard J.H. (2004). Moving toward a more patient-centered health care delivery system. Health Aff..

[bb0065] Kivi M., Eriksson M.C.M., Hange D., Petersson E.L., Björkelund C., Johansson B. (2015). Experiences and attitudes of primary care therapists in the implementation and use of internet-based treatment in swedish primary care settings. Internet Interv..

[bb0070] Kloek C.J.J., Bossen D., de Bakker D.H., Veenhof C., Dekker J. (2017). Blended interventions to change behavior in patients with chronic somatic disorders: systematic review. J. Med. Internet Res..

[bb0075] Kloek C.J.J., Bossen D., Spreeuwenberg P.M., Dekker J., de Bakker D.H., Veenhof C. (2018). Effectiveness of a blended physical therapist intervention in people with hip osteoarthritis, knee osteoarthritis, or both: a cluster- randomized controlled trial. Phys. Ther..

[bb0080] Kroenke K., Jackson J.L. (1998). Outcome in general medical patients presenting with common symptoms: a prospective study with a 2-week and a 3-month follow-up. Fam. Pract..

[bb0085] Kwasnicka D., Dombrowski S.U., White M., Sniehotta F. (2016). Theoretical explanations for maintenance of behaviour change: a systematic review of behaviour theories. Health Psychol. Rev..

[bb0090] Landelijke Stuurgroep Multidisciplinaire Richtlijnontwikkeling in de GGZ (2010). Somatisch Onvoldoende verklaarde Lichamelijke Klachten (SOLK) en Somatoforme Stoornissen.

[bb0095] Lentferink A.J., Oldenhuis H.K.E., De Groot M., Polstra L., Velthuijsen H., Van Gemert-Pijnen J.E.W.C. (2017). Key components in eHealth interventions combining self-tracking and persuasive eCoaching to promote a healthier lifestyle: a scoping review. J. Med. Internet Res..

[bb0100] Meijer W.M., Verberne L.D.M., Weesie Y.M., Davids R.N., Winckers M.L.J.J., Korteweg L., Hek K. (2019). Zorg door de huisarts.

[bb0105] Olde Hartman T.C., Borghuis M.S., Lucassen P.L., van de Laar F.A., Speckens A.E., van Weel C. (2009). Medically unexplained symptoms, somatisation disorder and hypochondriasis: course and prognosis. A systematic review. J. Psychosom. Res..

[bb0110] Patel S., Akhtar A., Malins S., Wright N., Rowley E., Young E., Morriss R. (2020). The acceptability and usability of digital health interventions for adults with depression, anxiety, and somatoform disorders: qualitative systematic review and meta-synthesis. J. Med. Internet Res..

[bb0115] Talboom-Kamp E.P., Verdijk N.A., Kasteleyn M.J., Numans M.E., Chavannes N.H. (2018). From chronic disease management to person-centered eHealth; a review on the necessity for blended care. Clin. EHealth.

[bb0120] Trimbos-instituut (2011). MDR SOLK en Somatorme Stoornissen.

[bb0125] van der Vaart R., Witting M., Riper H., Kooistra L., Bohlmeijer E.T., van Gemert-Pijnen L.J.E.W.C. (2014). Blending online therapy into regular face-to-face therapy for depression: content, ratio and preconditions according to patients and therapists using a Delphi study. BMC Psychiatry.

[bb0130] van Dessel N., den Boeft M., van der Wouden J.C., Kleinstäuber M., Leone S.S., Terluin B., van Marwijk H. (2014). Non-pharmacological interventions for somatoform disorders and medically unexplained physical symptoms (MUPS) in adults (review). Cochrane Database Syst. Rev..

[bb0135] van Ewijk C., van der Horst A., Besseling P. (2013). Toekomst voor de zorg. Gezondheid loont. Tussen keuze en solidariteit.

[bb0140] van Gemert-Pijnen J.E.W.C., Nijland N., van Limburg M., Ossebaard H.C., Kelders S.M., Eysenbach G., Seydel E.R. (2011). A holistic framework to improve the uptake and impact of eHealth technologies. J. Med. Internet Res..

[bb0145] van Westrienen P.E., Pisters M.F., Gerrits M., Veenhof C., de Wit N.J. (2019). Identifying treatment modalities for a multidisciplinary and blended care intervention for patients with moderate medically unexplained physical symptoms: qualitative study among professionals. JMIR Ment. Health.

[bb0150] van Westrienen P.E., Pisters M.F., Toonders S.A., Gerrits M., Veenhof C., de Wit N.J. (2018). Effectiveness of a blended multidisciplinary intervention for patients with moderate medically unexplained physical symptoms (PARASOL): protocol for a cluster randomized clinical trial. JMIR Res. Protocol..

[bb0155] Van Westrienen P.E., Pisters M.F., Toonders S.A.J., Gerrits M., Veenhof C., De Wit N.J. (2018). Effectiveness of a blended multidisciplinary intervention for patients with moderate medically unexplained physical symptoms (PARASOL): protocol for a cluster randomized clinical trial. J. Med. Internet Res..

[bb0160] Van Westrienen P.E., Pisters M.F., Veenhof C., De Wit N.J. (2019). Identification of patients with moderate medically unexplained physical symptoms in primary care with a five years follow-up. BMC Fam. Pract..

[bb0165] Verhaak P.F.M., Meijer S.A., Visser A.P., Wolters G. (2006). Persistent presentation of medically unexplained symptoms in general practice. Fam. Pract..

[bb0170] Volker D., Zijlstra-Vlasveld M.C., Anema J.R., Beekman A.T.F., Brouwers E.P.M., Emons W.H.M., Van der Feltz-Cornelis C.M. (2015). Effectiveness of a blended web-based intervention on return to work for sick-listed employees with common mental disorders: results of a cluster randomized controlled trial. J. Med. Internet Res..

[bb0175] Wentzel J., van der Vaart R., Bohlmeijer E.T., van Gemert-Pijnen J.E.W.C. (2016). Mixing online and face-to-face therapy: how to benefit from blended Care in Mental Health Care. JMIR Ment. Health.

[bb0180] Wouters M., Swinkels I., Lettow B., de Jong J., Sinnige J., Brabers A., van Gennip L. (2018). E-health in verschillende snelheden; eHealth-monitor 2018.

[bb0185] Zeylemaker M.M.P., Linn F.H.H., Vermetten E. (2015). Blended care; development of a day treatment program for medically unexplained physical symptoms (MUPS) in the dutch armed forces. Work.

